# Clinical Outcomes after Complete Intracorneal Ring Implantation and Corneal Collagen Cross-Linking in an Intrastromal Pocket in One Session for Keratoconus

**DOI:** 10.1155/2014/568128

**Published:** 2014-09-08

**Authors:** Pavel Studeny, Deli Krizova, Zbynek Stranak

**Affiliations:** Ophthalmology Department, 3rd Medical Faculty, Charles University and University Hospital Kralovske Vinohrady, Srobarova 50, 10034 Prague, Czech Republic

## Abstract

*Purpose.* The aim of this work was to evaluate the results after combined surgery implantation of full rings and CXL in one session in a group of patients with keratoconus during a 12-month follow-up. *Material and Methods.* The study included 22 eyes of 20 keratoconic patients, mean age of 28.41 (from 18 to 50) years. A full ring was inserted and afterwards 0.1% riboflavin solution was injected into the corneal pocket through the incision tunnel. The cornea was irradiated with UV-A light for 30 minutes. Postoperative visits were scheduled for the first week and months 1, 3, 6, 12, and 24 after surgery. Minimal follow-up time was 12 months. *Results.* The mean UDVA improved by 6 lines from before the operation to 1 year after the operation, the mean CDVA improved by approximately 2.5 lines, and the mean K improved by 3.94 D. Statistically significant reductions of sphere (*P* < 0.001), cylinder (*P* = 0.004), and spherical (*P* < 0.001) equivalents were found 1 month after surgery. *Conclusion.* The combined surgery MyoRing implantation and CXL seems to be a safe method in the treatment of keratoconus. We noticed an improvement of the refractive error in all of our patients.

## 1. Introduction

Keratoconus is an ectatic corneal disorder with progressive steepening and corneal thinning, especially in the inferior part of the cornea. Ultraviolet-A irradiation of the cornea after the application of riboflavin induces cross-links between the collagen elements with subsequent stiffening of the tissue [1]. Although this treatment may stop the progression of keratoconus and stabilise the cornea, the ability to achieve visual rehabilitation for improved visual outcome is limited [2, 3].

Corneal remodelling by inserting intrastromal implants can improve the visual acuity, changing the curvature of the ectatic cornea [4]. Incomplete rings available in the market for many years are Intacs (Addition Technology, Inc.), Ferrara ring (Ferrara Ophthalmics Ltd.), and Keraring (Mediphacos Ltd.). Implanting of a complete intrastromal ring, MyoRing (Dioptex GmbH, Austria), is an alternative technique, which has been proven to be safe and effective in previous studies in the treatment of keratoconus [5–9]. The ring is implanted into an intrastromal pocket created with either a specified microkeratome PocketMaker (Dioptex GmbH, Austria) [5] or a femtosecond laser [8, 10]. The depth of the corneal pocket has been proposed to be 300 or 250 *μ*m in the previous studies [5, 11]. The main advantages of a full ring are easy implantation, excellent centration, and the postoperative possibility of adjusting the position of the ring, if necessary [6]. The corneal pocket can also be used for the direct application of the riboflavin into the cornea. Bypassing the epithelium by injecting riboflavin directly into an intracorneal pocket seems to be a safe and effective method, preserving the epithelium and avoiding pain and discomfort seen after epithelial removal [12]. The combination of full ring implantation and corneal cross-linking (CXL) not only can lead to an improvement but also can lead to a long-term stability of visual acuity in patients with keratoconus [12].

The aim of this work is to evaluate the results after combined surgery implantation of full rings and CXL in one session in a group of patients with keratoconus during a 12-month follow-up.

## 2. Materials and Methods

This was a retrospective, consecutive, nonrandomised, interventional case series including a total of 22 eyes of 20 keratoconic patients with ages ranging from 18 to 50 years. Informed consent was obtained from all patients. Institutional ethical review board approval was obtained for the procedures and the tenets of the Helsinki Declaration were followed.

Keratoconus diagnosis was based on corneal topography and slit-lamp observation: asymmetric bow tie pattern, the presence of stromal thinning, conical protrusion of the cornea at the apex, Fleischer ring, and Vogt striae. Patients were classified according to the Amsler-Krumeich classification [13]. Inclusion criteria were keratoconic eyes with no corneal scar, minimum corneal thickness 350 *μ*m, and uncorrected distance visual acuity (UDVA) worse than 0.25 logMAR. Exclusion criteria were active ocular diseases, history of herpes keratitis, hyperopic spherical equivalent (SE), previous intraocular or corneal surgery, systemic connective tissue disease, and pregnancy.

All surgical procedures were performed by 1 surgeon (P.S.). After topical anesthesia a closed intracorneal pocket was created via a small incision tunnel by means of the PocketMaker microkeratome. The diameter of the pocket was 9.0 mm and the depth was 300 *μ*m. The incision tunnel was approximately 4.0 mm wide and 2.0 mm long. A detailed description of the creation of the corneal pocket using a microkeratome was described by Daxer [14]. The MyoRing was then inserted into the pocket. The diameters of the rings used in this study were 5 or 6 mm with a thickness of 240, 280, or 320 *μ*m, according to the nomogram recommended by the manufacturer. A sterile standard dose of riboflavin without dextran (0.1% riboflavin, Mediocross-sine, Medio-HAUS Medizinprodukte GmbH, Germany) was continuously injected over 1 minute into the corneal pocket via a standard cannula of 0.3 mm diameter through the incision tunnel. The instillation of the dye resulted in a yellowish colour of the anterior and posterior stroma, visible in the slit-lamp microscopy. The cornea was irradiated with UV-A light of 365 nm (Peschke Meditrade GmbH, Switzerland) and UV intensity of 3 mW/cm^2^ for 30 minutes. The intracorneal tunnel is self-sealing, and the procedure requires no suturing.

Preoperatively and at all postoperative visits, patients had a complete ocular examination. The examination included UDVA, corrected distance visual acuity (CDVA), manifest refraction, slit lamp microscopy, and Pentacam imaging (Oculus GmbH, Germany). The primary outcome measures were the safety of the procedure, defined as the number and percentage of eyes losing more than 2 lines of Snellen UDVA, the safety index, defined as mean postoperative CDVA/mean preoperative CDVA [15], the UDVA and CDVA, manifest refractions, and keratometry. Keratometry and corneal thickness were measured using the Pentacam Scheimpflug imaging system. The UDVA and CDVA were obtained in decimal scaling and transformed into logMAR for statistical analysis.

Postoperative visits were scheduled for the first week and months 1, 3, 6, 12, and 24 after surgery. The minimal follow-up time was 12 months and 11 eyes had a follow-up time of 24 months.

Preoperative data versus postoperative data were analysed using the paired *t*-test. Statistical measures are the mean ± standard deviation and significant *P* values are less than 0.05. Statistical analysis was performed using SPSS statistic software, version 15.0, for Windows (SPSS, Inc., IL, USA).

## 3. Results

A total of 22 eyes of 20 patients with a mean age of 28.41 (±8.94) years were included; 14 patients were male (70%) and 6 were female (30%). According to the Amsler-Krumeich grading system 4 eyes had a keratoconus grade I (18.18%), 7 eyes had a keratoconus grade II (31.82%), and 11 eyes had a keratoconus grade III (50%). No intraoperative complications occurred. No MyoRing was explanted after surgery. Only five eyes had a temporary slight haziness of the cornea, which completely disappeared within one week. We have not noticed any serious postoperative complications. One patient recorded deterioration in UDVA from 0.2 to 0.05 1 month after surgery and 6 months after surgery UDVA returned to the original value of 0.2. The safety index was 1.7 at 1 year.

**Figure 1:**
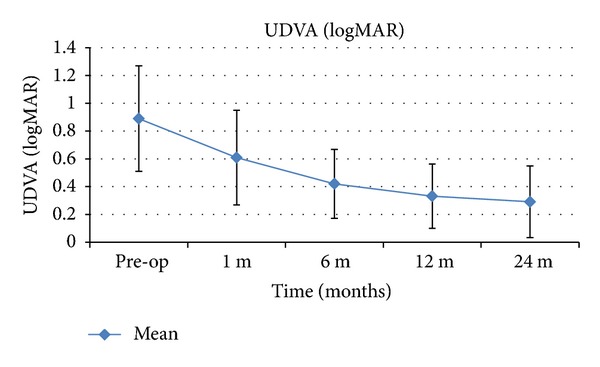
Mean UDVA over time. The error bars represent the SD in logMAR (UDVA: uncorrected distance visual acuity).

**Figure 2:**
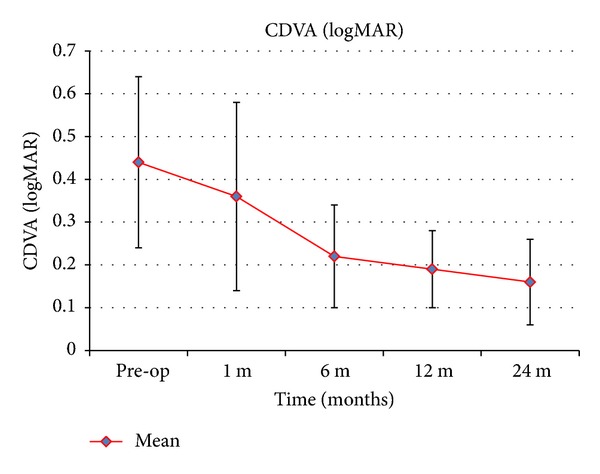
Mean CDVA over time. The error bars represent the SD in logMAR (CDVA: corrected distance visual acuity).

Table 1 summarises the visual and refractive outcomes over time. A significant improvement in UDVA was observed 1 month after surgery (*P* = 0.014). We noticed further improvement in subsequent periods (Figure 1). The difference between the first month and the sixth month was statistically significant (*P* = 0.011) and the difference between the sixth month and the twelfth month was not statistically significant (*P* = 0.227). Statistically significant reductions of sphere (*P* < 0.001), cylinder (*P* = 0.004), and spherical (*P* < 0.001) equivalents were found 1 month after surgery. No significant changes in manifest refraction were detected during the remaining follow-up. The improvement in CDVA 1 month after surgery was not statistically significant (*P* = 0.243) but we noticed further increasing in subsequent periods (Figure 2). The difference between the first month and the sixth month was statistically significant (*P* = 0.001) and the difference between the sixth month and the twelfth month was not significant (*P* = 0.209).

**Figure 3:**
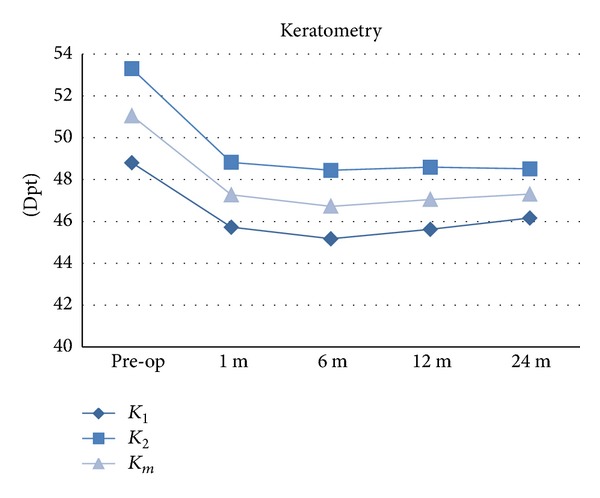
Keratometric changes after MyoRing implantation and CXL. D: diopters; *K*_1_: corneal dioptric power in the flattest meridian for the 3 mm central zone; *K*_2_: corneal dioptric power in the steepest meridian for the 3 mm central zone; *K*_*m*_: mean corneal power in the 3 mm central zone.

Regarding corneal topographic outcomes (Table 2 and Figure 3) there was significant central corneal flattening (mean keratometry) 1 month after surgery (*P* < 0.001). However further improvement was no longer statistically significant (between 1 month and 6 months, *P* = 0.191, and between 6 months and 1 year, *P* = 0.502). Also, the mean value of corneal astigmatism (keratometry in flat meridian-keratometry in steep meridian) decreased significantly only in the first month after operation (*P* = 0.031).

**Figure 4:**
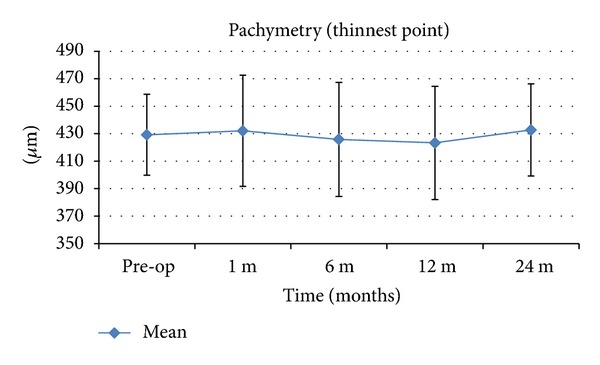
Pachymetric changes after MyoRing implantation and CXL. *μ*m: micrometer. The error bars represent the SD in *μ*m.

In the postoperative period, we did not notice any thinning of the cornea and the preoperative and postoperative differences in the mean thinnest corneal point were not statistically significant. Preoperatively, the pachymetry was 429.18 ± 29.47 *μ*m and 1 month postoperatively it was 432.09 ± 40.79 *μ*m (*P* = 0.576) and 1 year postoperatively it was 423.29 ± 41.23 *μ*m (*P* = 0.210) (Table 2 and Figure 4).

## 4. Discussion

Many clinical studies have demonstrated the effectiveness of CXL to stop the progression of keratoconus [2, 16, 17]. The CXL causes photopolymerisation of collagen fibrils in the corneal stroma and it subsequently modifies the biomechanical properties of the cornea, especially the resistance to stretching [18, 19]. The main disadvantage of standard CXL with removal of epithelium is a greater risk of infection and pain. The method of CXL without removing the epithelium was therefore proposed. In some studies, however, the effect of transepithelial CXL has been proven as limited in terms of biomechanical and functional efficacies [20, 21]. In 2009, Kanellopoulos described the technique of CXL with the intrastromal application of riboflavin into the pocket created by femtosecond laser [22].

More recently, techniques combining intrastromal corneal ring segment and CXL with the intrastromal administration of riboflavin have been described. A theoretical advantage of this method is the combination of two effects onto the ectatic cornea. Alió et al. compared 2 techniques of CXL using an epithelial debridement or intrastromal pocket technique after previous corneal ring segment implantation in eyes with keratoconus. They report that CXL with intrastromal riboflavin injection seemed to be as effective for corneal and refractive changes as classic CXL, although with potentially less postoperative pain [23]. Also Kılıç et al., in their study of 131 eyes with keratoconus, treated by CXL with a riboflavin injection into the corneal channel, combined with intrastromal corneal ring segment implantation, concluded that this technique is effective and the intrastromal riboflavin injection into the tunnel is safe and may provide more penetration without epithelial removal [24]. But, there may be one potential risk and disadvantage. The tunnel for segment implantation and riboflavin injection is relatively narrow and is located in the middle periphery of the cornea so the saturation of the central part of the cornea with riboflavin may not be absolutely perfect. Daxer et al. described the technique of MyoRing implantation and CXL with the intrastromal application of riboflavin into the pocket in one session. Authors presented one case report with a very good result. UDVA increased by 7 lines from 0.05 to 0.25, and the average central *K* reading decreased by 11 diopters. They noticed corneal haze during the early postoperative period. It diminished in the first month after surgery [12].

In our work, we evaluated the annual results of combined treatment with the intrastromal CXL application of riboflavin and full corneal ring implantation in a group of 22 eyes with keratoconus. One month after surgery we noticed a statistically significant improvement in all the followed parameters. The mean UDVA increased from 0.89 logMAR to 0.61 logMAR, mean CDVA from 0.44 logMAR to 0.36 logMAR, mean *K* from 51.05 D to 47.27 D, mean sphere from −4.01 D to −1.54 D, and mean cylinder from −2.98 D to −1.15 D and similar improvements have also been described by Jabbarvand et al. and Alio et al. They implanted only MyoRing, without the use of CXL. Jabbarvand et al. in a group of 98 eyes, describe, one month after MyoRing implantation, an improvement of the mean UDVA from 1.17 logMAR to 0.66 logMAR, mean CDVA from 0.85 logMAR to 0.51 logMAR, mean *K* from 51.9 D to 45.0 D, mean sphere from −5.48 D to 0.08 D, and mean cylinder from −5.3 D to −2.21 D. Between 1 month and 12 months after implantation monitored parameters have remained unchanged, or they changed only slightly. 1 year after surgery the mean UDVA was 0.62 logMAR, mean CDVA was 0.52 logMAR, mean K was 45.0 D, mean sphere was 0.09 D, and mean cylinder was −2.23 D [10]. 1 month after MyoRing implantation in a group of 12 eyes Alio et al. described an improvement of the mean UDVA from 1.36 logMAR to 0.69 logMAR, mean cylinder from −6.75 D to −2.07 D, and mean sphere from −4.82 D to −0.5 D. CDVA remains unchanged, 0.1 logMAR. As in the previous study, the results one year after surgery compared with results one month after surgery remained almost unchanged [8].

In our work we noticed a further improvement of the results between 1 month and 1 year after surgery. One year after surgery UDVA was 0.33 logMAR, CDVA 0.19 logMAR, mean K 47.11 D, mean sphere −1.37, and mean cylinder −1.37 D. Improvement in UDVA and CDVA was statistically significant (*P* = 0.008; *P* = 0.011, resp.).

After CXL, a slight improvement in long-term follow-up period is a common finding [2]. In contrast, after implantation of the rings, the findings are stable after 1 month and do not change. It can be assumed that the slight improvement of followed parameters a year after surgery can be attributed to the effect of CXL only.

## 5. Conclusion

The combined surgery MyoRing implantation and CXL seems to be a safe method in the treatment of keratoconus. We noticed an improvement of the refractive error in all of our patients. The exact resolution between the effects of CXL with intrastromal submitted riboflavin and MyoRing implantation will require additional studies with a longer follow-up period.

## Figures and Tables

**Table 1:** Visual and refractive outcomes over time.

Mean ± SD						
Variable	Preoperative	Postoperative				*P* Value
		1 Months	6 Months	1 Year	2 Years
UDVA (logMAR)	0.89 ± 0.38	0.61 ± 0.34	0.42 ± 0.26	0.33 ± 0.23	0.29 ± 0.26	=0.012
CDVA (logMAR)	0.44 ± 0.20	0.36 ± 0.22	0.22 ± 0.12	0.19 ± 0.09	0.16 ± 0.10	<0.001
Sphere (D)	−4.01 ± 3.21	−1.54 ± 2.57	−0.94 ± 2.07	−1.25 ± 0.94	−1.8 ± 0.58	<0.001
Cylinder (D)	−2.98 ± 2.67	−1.15 ± 1.78	−1.53 ± 2.10	−1.37 ± 1.43	−0.95 ± 1.54	=0.025
Mean K (D)	51.05 ± 4.51	47.27 ± 5.27	46.8 ± 4.64	47.11 ± 4.57	47.34 ± 5.85	<0.001
Corneal astigmatism (D)	4.62 ± 3.23	3.12 ± 1.92	1.66 ± 0.9	2.96 ± 1.65	2.35 ± 1.14	=0.048

CDVA = corrected distance visual acuity; K = keratometry; UDVA = uncorrected distance visual acuity.

*P* value—change from preoperatively to 1 year postoperatively (paired Student *t* test).

**Table 2:** Keratometry (D) and pachymetry (*µ*m) over time.

Mean ± SD						
Variable	Preoperative	Postoperative				*P* Value
		1 Months	6 Months	1 Year	2 Years	
*K*_1_	48.80 ± 4.68	45.72 ± 5.37	45.17 ± 4.72	45.62 ± 4.55	46.16 ± 5.78	<0.001
*K*_2_	53.30 ± 4.95	48.82 ± 5.35	48.44 ± 4.74	48.59 ± 4.75	48.51 ± 5.98	<0.001
*K*_*m*_	51.05 ± 4.52	47.27 ± 5.28	46.72 ± 4.63	47.05 ± 4.57	47.3 ± 5.85	<0.001
Pachymetry (thinnest location)	429.18 ± 29.47	432.09 ± 40.49	425.80 ± 41.50	423.29 ± 41.23	432.73 ± 33.60	=0.210

D = diopters; *µ*m = micrometres; *K*_1_ = corneal dioptric power in the flattest meridian for the 3-mm central zone, *K*_2_ = corneal dioptric power in the steepest meridian for the 3-mm central zone, *K*_*m*_ = mean corneal power in the 3-mm central zone.

*P* value—change from preoperatively to 1 year postoperatively (paired Student *t* test).
